# Role of *SIRT1* Gene Polymorphisms and Serum Levels in Patients with Multiple Sclerosis

**DOI:** 10.3390/diagnostics13203287

**Published:** 2023-10-23

**Authors:** Kriste Kaikaryte, Greta Gedvilaite, Renata Balnyte, Ingrida Uloziene, Rasa Liutkeviciene

**Affiliations:** 1 Laboratory of Ophthalmology, Neuroscience Institute, Medical Academy, Lithuanian University of Health Sciences, Eiveniu 2, 50161 Kaunas, Lithuania; greta.gedvilaite@lsmuni.lt (G.G.); rasa.liutkeviciene@lsmuni.lt (R.L.); 2 Department of Neurology, Medical Academy, Lithuanian University of Health Sciences, Eiveniu 2, 50161 Kaunas, Lithuania; renata.balnyte@lsmuni.lt; 3 Department of Otorhinolaryngology, Lithuanian University of Health Sciences, 44307 Kaunas, Lithuania; ingrida.uloziene@lsmuni.lt; 4 Department of Ophthalmology, Medical Academy, Lithuanian University of Health Sciences, Eiveniu 2 Str., 50161 Kaunas, Lithuania

**Keywords:** multiple sclerosis, *SIRT1*, SIRT1 ELISA, SIRT1 SNP

## Abstract

Aim: The purpose of this work was to investigate the prevalence of *SIRT1* rs3818292, rs3758391, and rs7895833 single nucleotide polymorphisms and SIRT1 serum levels associated with multiple sclerosis (MS) in the Lithuanian population. Methods: A total of 250 MS patients and 250 healthy controls were included in the study. Genotyping was performed using the RT-PCR method. Statistical analysis was performed using “IBM SPSS version 29.0”. The serum SIRT1 level was determined by the ELISA method. Results: We found that rs3818292 was associated with increased odds of developing MS under the dominant (*p* = 0.007) and allelic genetic (*p* = 0.004) models. rs3758391 was associated with increased odds of developing under the co-dominant (*p* < 0.001), overdominant (*p* < 0.001), dominant (*p* < 0.001), and allelic (*p* = 0.002) genetic models. rs7895833 was associated with increased odds of developing MS under co-dominant (*p* < 0.001), overdominant (*p* < 0.001), dominant (*p* < 0.001), and allelic (*p* < 0.001) genetic models. Additional sex-differentiated analysis within females revealed that the rs3758391 was associated with an increased odds ratio for the occurrence of MS among the co-dominant (*p* = 0.006), dominant (*p* = 0.002), and allelic (*p* = 0.001). rs7895833 was associated with an increased odds ratio for the development of MS under the co-dominant (*p* < 0.001), overdominant (*p* < 0.001), dominant (*p* < 0.001), and allelic (*p* < 0.001) genetic models. Age-differentiated analysis showed that rs3758391 was associated with an increased odds ratio for the development of MS in younger patients under the codominant (*p* = 0.002), overdominant (*p* = 0.003), and dominant (*p* = 0.004) genetic models. rs7895833 was associated with an increased odds ratio for the occurrence of MS under the overdominant genetic model (*p* = 0.013). In elderly patients, rs3818292 was associated with an increased odds ratio for the occurrence of MS under the dominant (*p* = 0.008) and allelic (*p* = 0.009) genetic models. rs7895833 was associated with an increased odds ratio for the occurrence of MS under the codominant (*p* = 0.011 and *p* = 0.012), dominant (*p* = 0.001), and allelic (*p* < 0.001) genetic models. We also found that serum SIRT1 levels were statistically significantly different between MS patients and control group subjects (*p* < 0.001). In addition, comparison of SIRT1 levels between study groups and genotypes showed that rs3818292 AA (*p* = 0.001), rs3758391 CT (*p* < 0.001), and rs7895833 AA (*p* = 0.002) and AG (*p* = 0.004) had higher SIRT1 levels in the control group than in the MS group. All results were provided after strict Bonferroni correction. Conclusions: Genetic variations in *SIRT1* rs3818292, rs3758391, and rs7895833 are associated with multiple sclerosis, with possible differences in gender and age, as well as lower serum SIRT1 levels.

## 1. Introduction

Multiple sclerosis (MS) is a chronic and unpredictable disease of the central nervous system characterized by the development of focal inflammatory lesions in the CNS that can cause a variety of neurological dysfunctions in early adulthood [1]. The exact cause of multiple sclerosis is unknown. It is thought to be a combination of genetic and environmental factors that trigger an autoimmune system. There is evidence that genetic factors may play a role in MS susceptibility [2]. Studies have shown that people with a family history of MS have a higher risk of developing the disease than people without this history. In addition, certain variations in genes involved in immune function have been associated with an increased risk of MS [3]. Regarding environmental factors, the incidence of MS varies greatly by region, race, age, and gender [4]. According to a systematic review, higher rates of MS have generally been reported in women and in populations living at higher latitudes, such as in Northern Europe, North America, and parts of Asia [5], as well as in Lithuania [6]. SIRT1 is a member of the sirtuin family of highly conserved III NAD class-dependent deacetylases involved in the regulation of cellular processes such as energy metabolism, DNA repair, aging, and inflammation [7]. Expression of SIRT1 has been detected in various mouse ocular tissues, including the cornea, lens, iris, ciliary body, inner nuclear layer, outer nuclear layer, and retinal ganglion cell layer [8]. In addition, SIRT1 is found in various neurons, including stem and progenitor cells, mature neurons, microglia, and astrocytes. SIRT1 is known to play a role in modulating immune response and reducing inflammation in various cell types, including immune cells in the central nervous system [9,10,11]. SIRT1 also controls neuronal development, axon growth, synaptic plasticity, and hormone secretion [12]. Both preclinical and clinical studies have shown that increasing the expression of SIRT1 can reduce autoimmunity as well as reduce the incidence of neurodegenerative diseases and neuroexcitation [13].

Although the exact mechanisms of SIRT1 in the pathogenesis of MS are not fully understood and are controversial, it is hypothesized that SIRT1 dysregulation may have an impact on the development and progression of MS. The impact of SIRT1 on this disease would be through its effects on immune function as well as oxidative stress, mitochondrial function, and autophagy networks [14]. In addition, SIRT1 can regulate inflammation by modulating the activation of master regulators such as NFkB and influencing antigen presentation by dendritic cells. The effects of SIRT1 on inflammation can be both anti-inflammatory and pro-inflammatory, and overexpression of SIRT1 improves symptoms in animal models of MS [15]. SIRT1 may also cooperate with Nrf2, a transcription factor involved in antioxidant production, mitochondrial biogenesis, and oxidative phosphorylation. Nrf2 has been linked to neurodegeneration and the pathogenesis of MS [16]. Studies have revealed the crucial role of SIRTs, including SIRT1, in the interaction between neuroinflammation, neurodegeneration, and metabolic changes [9], and SIRT1 has been implicated in the pathogenesis of several neurological diseases such as Alzheimer’s disease, Parkinson’s disease, and Huntington’s disease [17,18]. As a result, SIRT1 has been identified as a potential therapeutic target for neurological diseases [19,20,21].

In this study, we investigated the potential association between *SIRT1* gene polymorphisms (rs3818292, rs3758391, and rs7895833) and serum levels of SIRT1 in patients with multiple sclerosis in Lithuania. The intronic rs3818292 variant can affect the gene splicing processes and rs3758391, together with rs7895833, are functional variants located in the promoter region [22,23,24]. We believe that those variants could lead to the altered SIRT1 protein expression.

## 2. Materials and Methods

### 2.1. Subjects and Ethical Statement

The study was conducted in accordance with the Declaration of Helsinki, and all participants gave informed consent. The study included 500 participants and was conducted in the Laboratory of Ophthalmology of the Neuroscience Institute of the Lithuanian University of Health Sciences. Participants were divided into two different groups:

### 2.2. Group I: Patients with Multiple Sclerosis (n = 250)

The selected 250 MS patients were treated in the clinics of LUHS (Lithuanian University of Health Sciences) in Kaunas between 1 January 2020 and 31 December 2023. The study included only patients with a confirmed MS diagnosis. MS diagnosis was made according to the widely accepted and revised McDonald criteria (2017) [25]. At the time of diagnosis, a lumbar puncture and CSF examination were performed. CSF samples were analyzed by isoelectric focusing and IgG-specific immunofixation to test for the presence of intrathecal specific OCBs. Demographic and clinical data and magnetic resonance imaging results were obtained from all patients. Disability was measured using the Kurtzke Expanded Disability Status Scale. Data were obtained from outpatient records, and retrospective analysis was performed. The following variables were considered in the selection process: patient age (at the time of diagnosis and first symptoms), gender, and disease progression [26].

Exclusion criteria for the study were systemic diseases such as diabetes mellitus, oncologic diseases, systemic tissue disorders, chronic infectious diseases, autoimmune diseases, and conditions after organ or tissue transplantation. 

### 2.3. Group II: Control Group (n = 250)

The control group included subjects who matched the age and sex of the MS group, had no history of autoimmune or neurologic disease, and were in good general health.

### 2.4. Polymorphism Selection

In this study, we aimed to investigate the relationship between MS and three specific genetic variations in *SIRT1*: rs3818292, rs3758391, and rs7895833. Our literature search revealed that although these specific polymorphisms have not been directly associated with multiple sclerosis, they have been linked to other diseases associated with the development of MS, such as autoimmune diseases and neurodegenerative diseases. *SIRT1* rs3818292 is known to be located in the intronic region, which has functional effects on gene expression and regulation [22,27]. *SIRT1* rs3758391 and rs7895833 are located in the promoter region that can affect gene expression [23,24]. Therefore, we decided to evaluate these specific polymorphisms as potential genetic risk factors for MS in our study.

The study also examined the distribution of genotypes and alleles of *SIRT1* rs3818292, rs3758391, and rs7895833 in MS and the control groups across different sexes and ages. For this purpose, we divided participants into two age groups based on the average age of the study population: those who were 40 years old or younger and those who were over 40 years old.

### 2.5. DNA Extraction, SIRT1 Genotyping, and SIRT1 Serum-Level Determination

DNA extraction and analysis of *SIRT1* rs3818292, rs3758391, and rs7895833 were performed in the Ophthalmology Laboratory of the Neuroscience Institute of the Lithuanian University of Health Sciences. DNA samples were obtained from venous blood using the DNA salting-out method. Briefly, venous blood samples (white blood cells) were collected and suspended in a buffer solution, followed by the addition of detergents to degrade cell membranes, proteinase K to hydrolyze proteins, and chloroform to deproteinize them. The DNA was then precipitated with ethanol.

TaqMan^®^ genotyping assays (Thermo Scientific, Pleasanton, CA, USA) were used to determine all single nucleotide polymorphisms (SNPs). Genotyping of *SIRT1* rs3818292, rs3758391, and rs7895833 was performed using a Step One Plus real-time PCR system (Applied Biosystems, Foster City, CA, USA) according to the manufacturer’s recommendations. Real-time PCR mixtures were prepared according to the appropriate protocol for SNP determination.

We added 1.5 μL of the samples’ DNA and 8.5 μL of the PCR reaction mixture to each of the 96 wells of the plate, along with the negative control. Real-time PCR was performed using the Allelic Discrimination program, and the assay was performed according to the manufacturer’s instructions. The program analyzed each genotype based on the fluorescence intensity of the different detectors (VIC and FAM).

Serum SIRT1 levels were measured in both control subjects and patients using a human SIRT1 enzyme-linked immunosorbent assay (ELISA) kit (Abcam, Cambridge, UK). Serum SIRT1 levels were measured in duplicate in 41 control subjects and 20 patients with MS.

The ELISA assay was performed according to the manufacturer’s instructions. Optical density at a wavelength of 450 nm was measured using a microplate reader (Multiskan FC microplate photometer, Thermo Scientific, Waltham, MA, USA). SIRT1 concentrations were calculated using the standard curve with a sensitivity range of 0.63–40 ng/mL and 132 pg/mL.

### 2.6. Statistical Analysis

Statistical analysis was performed with SPSS/W 29.0 software (IBM Corp, Armonk, NY, USA). Sex distribution was presented in absolute numbers and percentages and compared with the chi-square test. Continuous data (age and serum SIRT1 level) were expressed as median with interquartile range (IQR). Data that were not normally distributed between the 2 groups or subgroups were compared with the Mann–Whitney U test.

We performed Hardy–Weinberg analysis with the χ^2^ test to analyze the observed and expected frequencies of *SIRT1* rs3818292, rs3758391, and rs7895833 in the control group. The analysis showed that all three SNPs met the HWE criteria (*p* > 0.05), indicating that the genotype and allele frequencies in the study were consistent with HWE expectations. We used the χ^2^ test to analyze the differences in the distribution of *SIRT1* rs3818292, rs3758391, and rs7895833 between the groups with MS and the control group. We also performed binary logistic regression analysis to evaluate the effects of genotypes on the development of multiple sclerosis, reporting odds ratios (OR) and 95% confidence intervals (CI). The best genetic model was selected based on the Akaike information criterion (AIC). According to the Akaike Information Criterion (AIC), the model with the lowest value is the most appropriate inheritance model. We considered statistically significant differences as those with *p* < 0.05 and adjusted our significance threshold for multiple comparisons to alpha = 0.017 (0.05/3, because we examined three SNPs in the *SIRT1* gene). Continuous data (age and serum SIRT1 level) were expressed as median with interquartile range (IQR) and compared between two groups or subgroups using the Mann–Whitney U test. Sex distribution was presented as absolute numbers with percentages and compared with the χ^2^ test.

## 3. Results

The study included a total of 500 subjects divided into two groups: 250 patients with MS and 250 control subjects. The control group was selected based on gender and age distribution to match the MS group. Females made up 65.5% (*n* = 164) of the MS group and 65.5% (*n* = 250) of the control group, while males made up 34.3% (*n* = 86) of the MS group and 34.3% (*n* = 86) of the control group (Table 1). 

In the MS group, the rs3818292 AA genotype and A allele were less common, whereas the AG genotype was more common compared with the control group (87.2% vs. 78.0%, *p* = 0.007; 93.0% vs. 87.0%, *p* = 0.002; and 11.6% vs. 18.0, *p* < 0.001, respectively). Similarly, the rs3758391 CC genotype and C allele were less common, whereas the CT genotype was more common in the MS group than in the control group (58.4% vs. 42.0%, *p* < 0.001; 75.8% vs. 67.2%, *p* = 0.003; and 34.8% vs. 50.4%, *p* < 0.001, respectively). Finally, the rs7895833 AA genotype and A allele were less common, whereas the AG genotype was more common in the MS group than in the control group (75.2% vs. 59.2%, *p* = <0.001; 21.2% vs. 34.8%, *p* = 0.001; and 85.8% vs. 76.6%, *p* < 0.001, respectively) (Table 2).

Our analysis revealed that individuals with the rs3818292 AG+GG genotype and each G allele had a 1.9-fold and 1.8-fold increased odds of developing MS under the dominant and allelic genetic models, respectively (OR = 1.921; CI: 1.193–3.095; *p* = 0.007 and OR = 1.806; CI: 1.203–2.711; *p* = 0.004, respectively). Similarly, the rs3758391 CT, CT+TT genotypes, and each T allele were associated with a 2-fold, 1.9-fold, 1.9-fold, and 1.6-fold increased odds of developing MS under the co-dominant, overdominant, dominant, and allelic genetic models, respectively (OR = 2.014; CI: 1.390–2.918; *p* < 0.001; OR = 1.904; CI: 1.329–2.727; *p* < 0.001; OR = 1.939; CI: 1.359–2.766; *p* < 0.001; and OR = 1.567; CI: 1.175–2.089; *p* = 0.002, respectively). Finally, individuals with the *SIRT1* rs7895833 AG, AG+GG genotypes, and each G allele had a 2.1-fold, 2-fold, 2.1-fold, and 1.8-fold increased odds of developing MS under the co-dominant, overdominant, dominant, and allelic genetic models, respectively (OR = 2.085; CI: 1.392–3.122; *p* < 0.001; OR = 1.984; CI: 1.330–2.959; *p* < 0.001; OR = 2.090; CI: 1.426–3.062; *p* < 0.001 and OR = 1.775; CI: 1.290–2.443; *p* < 0.001, respectively) (Table 3).

The findings of genotypes and alleles of *SIRT1* rs3818292, rs3758391, and rs7895833 in MS and control groups between different gender distributions suggest that, in women, the *SIRT1* rs3758391 CC genotype and each C allele were less frequent in those with MS compared with the control group (62.8% vs. 45.7%, *p* = 0.002; and 79.9% vs. 69.2% *p* = 0.002, respectively). The rs7895833 AA genotype and A allele were less common, whereas the AG genotype was more common in the MS group than in the control group (78.0% vs. 59.1%, *p* < 0.001; 87.8% vs. 77.7%, *p* = 0.001; and 19.5% vs. 37.2%, *p* < 0.001; respectively). Regarding men, the results showed that the rs3758391 CT genotype was more common in those with MS than in the control group (36.0% vs. 57.0%, *p* = 0.006) (Table 4).

A binary logistic regression analysis within different genders indicated that the *SIRT1* gene rs3758391 CT and CT+TT genotypes, as well as each T allele, were significantly associated with an increased odds ratio of MS occurrence under the co-dominant, dominant, and allelic genetic models. Specifically, the odds ratios were 1.9-fold, 2-fold, and 1.9-fold, respectively (OR = 1.888; CI: 1.198–2.976; *p* = 0.006; OR = 2.004; CI: 1.289–3.115; *p* = 0.002 and OR = 1.859; CI: 1.273–2.716; *p* = 0.001).

Similarly, the rs7895833 AG and AG+GG genotypes, as well as each G allele, were also significantly associated with an increased odds ratio of developing MS under the co-dominant, overdominant, dominant, and allelic genetic models. The odds ratios were 2.5-fold, 2.4-fold, 2.5-fold, and 2.1-fold, respectively (OR = 2.515; CI: 1.522–4.158; *p* < 0.001; OR = 2.443; CI: 1.483–4.025; *p* < 0.001; OR = 2.456; CI: 1.515–3.982; *p* < 0.001, and OR = 2.079; CI: 1.352–3.195; *p* < 0.001) (Table 5). However, there was no significant difference observed among men.

The results of genotypes and alleles of *SIRT1* rs3818292, rs3758391, and rs7895833 in MS and control groups between different ages (40 or younger, and over 40 years old) indicated that the rs3758391 CC genotype was less frequent, whereas that of the CT genotype was higher in the MS group than in the control group in the younger participants (56.7% vs. 38.6%, *p* = 0.004; and 35.0% vs. 53.8%, *p* = 0.003, respectively). The frequency of the rs7895833 AG genotype was also higher in the MS group than in the control group (23.3% vs. 37.9%; *p* = 0.012) (Table 6).

However, in the older participants, the frequency of the rs3818292 AA genotype and A allele was lower than in the control group (89.2% vs. 76.3% and 93.8% vs. 85.6%; *p* = 0.007 and *p* = 0.002, respectively), and the frequency of the rs7895833 AA genotype and A allele was also lower than in the control group (78.5% vs. 59.3%, *p* = 0.001; and 88.1% vs. 75.0%, *p* < 0.001; respectively) (Table 7).

Binary logistic regression analysis in younger patients revealed that *SIRT1* rs3758391 CT and CT+TT genotypes were associated with a 2.3-fold, 2.2-fold, and 2.1-fold increased odds of MS occurrence under the co-dominant, overdominant, and dominant genetic models (OR = 2.254; CI: 1.331–3.443; *p* = 0.002; OR = 2.162; CI: 1.301–3.592; *p* = 0.003 and OR = 2.077; CI: 1.256–3.435; *p* = 0.004, respectively). rs7895833 AG genotype was associated with a 2–fold increased odds of MS occurrence under the overdominant genetic model (OR = 2.003; CI: 1.156–3.473; *p* = 0.013) (Table 8). 

Moreover, in older patients, *SIRT1* rs3818292 AG+GG genotypes and each G allele were associated with a 2.6-fold and 2.2-fold increased odds of MS occurrence under the dominant and allelic genetic models (OR = 2.578; CI: 1.282–5.181; *p* = 0.008; OR = 2.177; CI: 1.219–3.890; *p* = 0.009, respectively). rs7895833 AG and GG genotypes were associated with a 2.2-fold and 5.3-fold increased odds of MS occurrence under the co-dominant genetic model (OR = 2.157; CI: 1.194–3.897; *p* = 0.011 and OR = 5.343; CI: 1.438–19.848; *p* = 0.012, respectively). Also, AG+GG genotypes and each G allele were associated with a 2.5-fold and 2.2-fold increased odds of MS occurrence under the dominant and allelic genetic models (OR = 2.498; CI: 1.432–4.358; *p* = 0.001 and OR = 2.225; CI: 1.403–3.528; *p* < 0.001, respectively) (Table 9).

### SIRT1 Serum Levels in Early and Multiple Sclerosis and Controls

**Figure 1:**
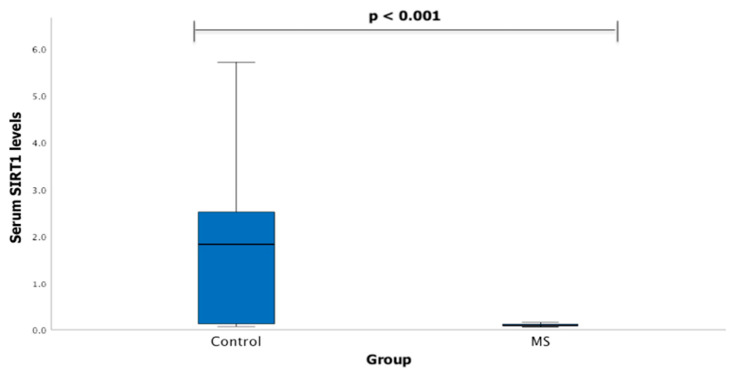
SIRT1 serum levels in patients with multiple sclerosis and control group subjects. Mann–Whitney U test was used to assess serum SIRT1 levels differences between patients with multiple sclerosis and control groups; *p* < 0.001.

Serum SIRT1 levels were measured in groups of patients with MS (*n* = 20) and healthy subjects (*n* = 41). We found that SIRT1 serum levels statistically significantly differ between MS patients and control group subjects (1.833 (2.488) ng/mL vs. 0.094 (0.038) ng/mL, *p* < 0.001) (Figure 1). 

**Figure 2:**
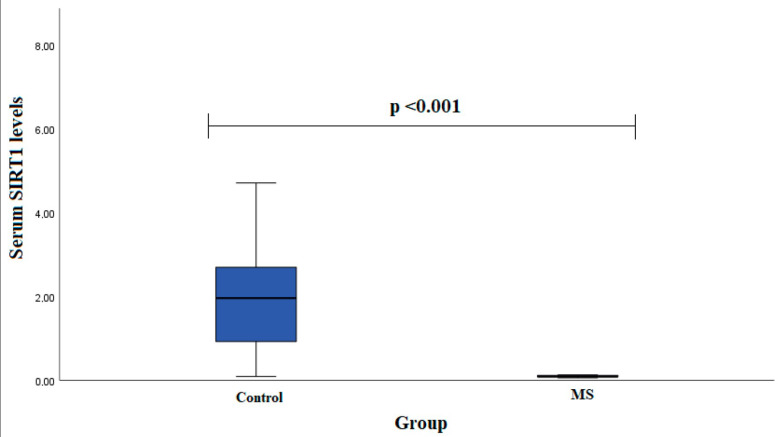
SIRT1 serum levels in patients with multiple sclerosis and control group subjects in female group. Mann–Whitney U test was used to assess serum SIRT1 levels differences between patients with multiple sclerosis and control groups; *p* < 0.001.

Serum SIRT1 levels were measured in groups of patients with MS (*n* = 13) and healthy subjects (*n* = 23). We found that females with MS had decreased SIRT1 serum levels compared to control group females (0.090 (0.047) ng/mL vs. 1.963 (2.614) ng/mL, *p* < 0.001) (Figure 2). 

**Figure 3:**
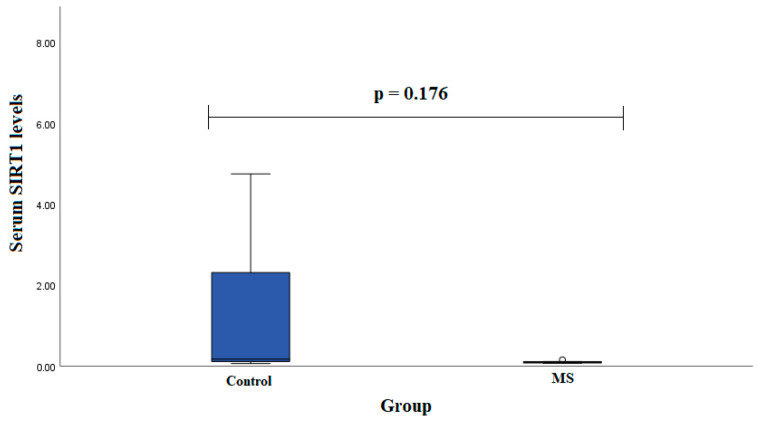
SIRT1 serum levels in patients with multiple sclerosis and control group subjects in male group. Student test was used to assess serum SIRT1 levels differences between patients with multiple sclerosis and control groups; *p* =0.176.

Serum SIRT1 levels were measured in groups of patients with MS (*n* = 7) and healthy subjects (*n* = 19). We found that SIRT1 serum levels did not statistically significantly differ between MS patients and control group subjects in males (0.102 (0.028) ng/mL vs. 2.291 (4.095) ng/mL, *p* < 0.001) (Figure 3). 

A serum SIRT1 level comparison between study groups and genotypes was performed and did show statistically significantly differences between two groups. We found that rs3818292 AA, rs3758391 CT, and rs7895833 AA and AG carriers had higher SIRT1 levels in the control group than the MS group (0.239 (2.377) vs. 1.245 (0.045); *p* = 0.001, 0.304 (2.770) vs. 0.089 (0.037); *p* < 0.001, 1.813 (2.812) vs. 0.094 (0.054); *p* = 0.002 and 1.872 (2.763) vs.0.089 (0.028); *p* = 0.004, respectively). A Mann–Whitney U test was used to compare SIRT1 levels between the two groups. The bars represent the median with the interquartile range (Table 10).

## 4. Discussion

In this study, we investigated the possible association between *SIRT1* gene polymorphisms and SIRT1 serum levels in patients with multiple sclerosis in Lithuania. We performed genotyping analysis of three specific single nucleotide polymorphisms (SNPs) within the *SIRT1* gene (rs3818292, rs3758391, and rs7895833). The analysis was performed on two groups consisting of 250 patients with MS and 250 control subjects. The results indicated an association between the three SNPs and a higher probability of developing MS.

As previously mentioned, gender is considered one of the risk factors for MS [4]. Our research results suggest that the variants of *SIRT1* gene, rs3758391 and rs7895833, are significantly associated with increased probability of developing MS. However, no significant difference was found in the results between males. Previous studies have shown that there are differences in the occurrence and clinical presentation of MS between genders [28]. Females tend to have an earlier onset of the disease MS and have a higher likelihood of relapse, while males tend to have a more aggressive form of the disease with faster progression of disability [29,30]. Hormonal factors may play a role in these sex differences, but the exact mechanisms are not fully understood [31].

Our research shows that the variants of the *SIRT1* gene, rs3758391 and rs7895833, are significantly associated with an increased likelihood of developing MS in younger patients, while the variants rs3818292 and rs7895833 are significantly associated with an increased likelihood of developing MS in older patients. It is known that age may also be a factor determining the prognosis of many neurodegenerative diseases, including MS [32]. The transition from the relapsing phase of MS, which is primarily inflammatory, to the secondary progressive phase of the disease, which is thought to be primarily neurodegenerative, is strongly associated with age and is considered the strongest predictor of this transition [33]. MS affects people of all ages but is most commonly diagnosed between 20 and 40 years of age [34]. The age of onset and clinical course of MS can vary widely, but patients with early-onset multiple sclerosis typically have relapsing-remitting disease, whereas patients with later-onset disease may experience more rapid progression to permanent disability [35].

Our study focused on specific genetic variations of *SIRT1* based on their location in the gene. The intronic variant rs3818292 may affect the splicing processes of the gene, while rs3758391 and rs7895833 are functional variants located in the promoter region [22,23,24,27]. These variants likely result in altered expression of the SIRT1 protein, as indicated by differences in serum levels between different groups and between carriers of different genotypes. We found that serum SIRT1 levels were higher in the control group than in the multiple sclerosis group. These results confirm previous conclusions that increasing SIRT1 expression can decrease autoimmunity and reduce the incidence of neurodegenerative disorders and neuroexcitation. To prevent neurological complications, it is critical to understand SIRT1 signaling and identify immune-mediated damage to neurons for potential therapeutic intervention [13].

The location of these *SIRT1* polymorphisms may play an important role in regulating gene expression and contribute to various disease susceptibilities.

Studies have shown that rs3818292 has a weak association with the risk of developing Parkinson’s disease (PD) [36]. Both diseases, PD and MS, affect the human nervous system [37]. In addition, mutation in the rs3818292 locus may be associated with a lower risk of developing diabetic kidney disease (DKD) [38]. The pathogenesis of kidney disease in patients with MS may be related to lower urinary tract dysfunction, recurrent urinary tract infections, and treatment with immunomodulatory agents such as interferons [39].

In addition, rs3818292 has been associated with visceral body fat in men with obesity [40]. Recent research has consistently shown that there is an association between obesity and an increased risk of developing multiple sclerosis [41].

Another *SIRT1* genetic variant, rs3758391, is a polymorphism that has been associated with various diseases such as type 2 diabetes, breast cancer, autoimmune thyroid disease, lupus erythematosus, and others [42].

Studies conducted with *SIRT1* rs7895833 are closely related to multiple sclerosis pathogenesis. There is an association between SIRT1 expression in the elderly and the rs7895833 variant in the *SIRT1* gene [43]. Another study showed that 42% of elderly patients in Brazil had variant allele G of the *SIRT1* gene polymorphism, which was associated with dyslipidemia [44]. It is well known that multiple sclerosis and dyslipidemia are linked through the association between inflammation and alterations in lipid metabolism [45]. In addition, an association between this polymorphism and increased risk of hypertension, higher body fat percentage, higher body mass index, and higher diastolic blood pressure has been demonstrated [46].

This study has shown significant associations between the genetic variations of *SIRT1* rs3818292, rs3758391, and rs7895833 and the development of multiple sclerosis, with possible differences in gender and age. In addition, these genetic variations were found to be associated with lower serum SIRT1 levels. Also, females with MS had decreased SIRT1 serum levels compared to control group females. These results suggest that genetic *SIRT1* variations may be potential prognostic factors for multiple sclerosis and may contribute to the identification of new therapeutic targets. However, further studies are needed to explore the precise mechanisms underlying the associations between genetic *SIRT1* variations and multiple sclerosis and to determine the generalizability of these findings to other populations.

This study is significant because it analyzes *SIRT1* rs3818292, rs3758391, and rs7895833, and serum SIRT1 levels in individuals with multiple sclerosis in the Lithuanian population and compares these results with those of healthy control subjects without other diseases, such as optic neuritis. Our study has some limitations to acknowledge. The relatively small sample size indicates the need for additional research with a larger cohort to draw stronger conclusions. Additionally, our analysis did not account for other potential risk factors like smoking, vitamin D levels, infection agents, and dietary preferences. Lastly, as our study exclusively focused on the Lithuanian population, its generalizability to other populations is limited. Despite these limitations, our findings offer valuable insights, serving as a foundation for future research and potential clinical applications. However, future studies should consider these limitations for a more comprehensive understanding.

## 5. Conclusions

Genetic variations in *SIRT1* rs3818292, rs3758391, and rs7895833 are associated with multiple sclerosis, with possible differences in sex and age, and lower serum SIRT1 levels.

## Figures and Tables

**Table 1:** Demographic characteristics of the study groups.

Characteristic	Group		*p*-Value
	Control Group	Multiple Sclerosis	
	*n* = 250	*n* = 250	
	*n* (%)	*n* (%)	
Gender			
Males	86 (34.4)	86 (34.4)	1.000
Females	164 (65.6)	164 (65.6)	
Age, years			
Mean (SD)	40 (12.4)	40 (9.9)	0.901

*p*-value—significance level and Bonferroni corrected significance level when *p* = 0.05/3.

**Table 2:** Distribution of genotypes and alleles of *SIRT1* rs3818292, rs3758391, and rs7895833 in the patients with multiple sclerosis and control groups.

Genotype/Allele	Control Group	Multiple Sclerosis	HWE	*p*-Value
	*n* = 250	*n* = 250	*p*-Value	
	*n* (%)	*n* (%)		
*SIRT1* rs3818292				
AA	218 (87.2) ^1^	195 (78.0) ^1^	0.085 *	**0.014**
AG	29 (11.6) ^2^	45 (18.0) ^2^		
GG	3 (1.2)	10 (4.0)		
A	465 (93.0)	435 (87.0)		**0.002**
G	35 (7.0)	65 (13.0)		
*SIRT1* rs3758391				
CC	146 (58.4) ^3^	105 (42.0) ^3^	0.416 *	**<0.001**
CT	87 (34.8) ^4^	126 (50.4) ^4^		
TT	17 (6.8)	19 (7.6)		
C	379 (75.8)	336 (67.2)		**0.003**
T	121 (24.2)	164 (32.8)		
*SIRT1* rs7895833				
AA	188 (75.2) ^5^	148 (59.2) ^5^	0.039 *	**<0.001**
AG	53 (21.2) ^6^	87 (34.8) ^6^		
GG	9 (3.6)	15 (6)		
A	429 (85.8)	383 (76.6)		**<0.001**
G	71 (14.2)	117 (23.4)		

*p*-value—significance level and Bonferroni corrected significance level when *p* = 0.05/3; ^1^ *p* = 0.007, ^2^ *p* < 0.001, ^3^ *p* < 0.001, ^4^ *p* < 0.001, ^5^ *p* < 0.001, ^6^ *p* = 0.001; * HWE criteria (*p* > 0.05) in the control group; the bolded results indicate significant differences between the groups.

**Table 3:** Binary logistic regression analysis of patients with multiple sclerosis and control groups.

Model	Genotype/Allele	OR (95% CI)	*p*-Value	AIC
Patients with Multiple Sclerosis				
*SIRT1* rs3818292				
Co-dominant	AG vs. AA	1.735 (1.047–2.875)	0.033	688.402
	GG vs. AA	3.726 (1.011–13.737)	0.048	
Dominant	AG+GG vs. AA	1.921 (1.193–3.095)	**0.007**	687.712
Recessive	GG vs. AG+AA	3.431 (0.933–12.618)	0.064	691.070
Overdominant	AG vs. AA+GG	1.673 (1.011–2.769)	0.045	691.059
Allelic	G	1.806 (1.203–2.711)	**0.004**	686.471
*SIRT1* rs3758391				
Co-dominant	CT vs. CC	2.014 (1.390–2.918)	<0.001	683.127
	TT vs. CC	1.554 (0.771–3.132)	0.218	
Dominant	CT+TT vs. CC	1.939 (1.359–2.766)	**<0.001**	681.639
Recessive	TT vs. CT+CC	1.127 (0.572–2.223)	0.729	695.027
Overdominant	CT vs. CC+TT	1.904 (1.329–2.727)	**<0.001**	682.650
Allelic	T	1.567 (1.175–2.089)	**<0.001**	685.569
*SIRT1* rs7895833				
Co-dominant	AG vs. AA	2.085 (1.392–3.122)	**<0.001**	682.518
	GG vs. AA	2.117 (0.901–4.973)	0.085	
Dominant	AG+GG vs. AA	2.090 (1.426–3.062)	**<0.001**	680.519
Recessive	GG vs. AG+AA	1.709 (1.734–3.982)	0.214	693.556
Overdominant	AG vs. AA+GG	1.984 (1.330–2.959)	**<0.001**	683.591
Allelic	G	1.775 (1.290–2.443)	**<0.001**	682.148

OR—odds ratio, AIC—Akaike information criteria; the underlined AIC value indicates the best genetic model; CI—confidence interval; *p*-value—significance level; Bonferroni corrected significance level when *p* = 0.05/3; the bolded results indicate significant differences between the groups.

**Table 4:** Distribution of genotypes and alleles of *SIRT1* rs3818292, rs3758391, and rs7895833 in MS and control groups between different genders.

Genotype/Allele	Control Group	Multiple Sclerosis	*p*-Value
	*n* = 250	*n* = 250	
	*n* (%)	*n* (%)	
Females			
*SIRT1* rs3818292			
AA	142 (86.6)	128 (78.0)	0.052
AG	22 (13.4)	33 (20.1)	
GG	0 (0)	3 (1.8)	
A	306 (93.3)	289 (88.1)	0.022
G	22 (6.7)	39 (11.9)	
*SIRT1* rs3758391			
CC	103 (62.8) ^1^	75 (45.7) ^1^	**0.005**
CT	56 (34.1)	77 (47.0)	
TT	5 (3)	12 (7.3)	
C	262 (79.9)	227 (69.2)	**0.002**
T	66 (20.1)	101 (30.8)	
*SIRT1* rs7895833			
AA	128 (78.0) ^2^	97 (59.1) ^2^	**0.001**
AG	32 (19.5) ^3^	61 (37.2) ^3^	
GG	4 (2.4)	6 (3.7)	
A	288 (87.8)	255 (77.7)	**0.001**
G	40 (12.2)	73 (22.3)	
Males			
*SIRT1* rs3818292			
AA	76 (88.4)	67 (77.9)	0.175
AG	7 (8.1)	12 (14.0)	
GG	3 (3.5)	7 (8.1)	
A	159 (92.4)	146 (84.9)	0.027
G	13 (7.6)	26 (15.1)	
*SIRT1* rs3758391			
CC	43 (50.0)	30 (34.9)	0.021
CT	31 (36.0) ^4^	49 (57.0) ^4^	
TT	12 (14.0)	7 (8.1)	
C	117 (68.0)	109 (63.4)	0.364
T	55 (32.0)	63 (36.6)	
*SIRT1* rs7895833			
AA	60 (69.8)	51 (59.3)	0.301
AG	21 (24.4)	26 (30.2)	
GG	5 (5.8)	9 (10.5)	
A	141 (82.0)	128 (74.4)	0.089
G	31 (18.0)	44 (25.6)	

*p*-value—significance level and Bonferroni corrected significance level when *p* = 0.05/3; ^1^ *p* = 0.002, ^2^ *p* < 0.001, ^3^ *p* < 0.001, ^4^ *p* = 0.006; the bolded results indicate significant differences between the groups.

**Table 5:** Binary logistic regression analysis of patients with multiple sclerosis and control groups between different genders.

Model	Genotype/Allele	OR (95% CI)	*p*-Value	AIC
Females				
*SIRT1* rs3818292				
Co-dominant	AG vs. AA	1.664 (0.922–3.002)	0.091	451.605
	GG vs. AA	-	-	
Dominant	AG+GG vs. AA	1.815 (1.015–3.248)	0.045	452.565
Recessive	GG vs. AG+AA	-	-	-
Overdominant	AG vs. AA+GG	1.626 (0.902–2.931)	0.106	454.046
Allelic	G	1.879 (1.083–3.261)	0.025	451.428
*SIRT1* rs3758391				
Co-dominant	CT vs. CC	1.888 (1.198–2.976)	**0.006**	447.982
	TT vs. CC	3.296 (1.114–9.753)	0.031	
Dominant	CT+TT vs. CC	2.004 (1.289–3.115)	**0.002**	447.024
Recessive	TT vs. CT+CC	2.511 (0.864–7.295)	0.091	453.577
Overdominant	CT vs. CC+TT	1.707 (1.094–2.664)	0.019	451.109
Allelic	T	1.859 (1.273–2.716)	**0.001**	445.996
*SIRT1* rs7895833				
Co-dominant	AG vs. AA	2.515 (1.522–4.158)	**<0.001**	444.822
	GG vs. AA	1.979 (0.544–7.208)	0.300	
Dominant	AG+GG vs. AA	2.456 (1.515–3.982)	**<0.001**	442.944
Recessive	GG vs. AG+AA	1.519 (0.421–5.486)	0.523	456.289
Overdominant	AG vs. AA+GG	2.443 (1.483–4.025)	**<0.001**	443.921
Allelic	G	2.079 (1.352–3.195)	**<0.001**	444.882

OR—odds ratio, AIC—Akaike information criteria; the underlined AIC value indicates the best genetic model; CI—confidence interval; *p*-value—significance level; Bonferroni corrected significance level when *p* = 0.05/3; the bolded results indicate significant differences between the groups.

**Table 6:** Distribution of genotypes and alleles of *SIRT1* rs3818292, rs3758391, and rs7895833 in the patients with multiple sclerosis and control groups between younger participants.

Genotype/Allele	Control Group	Multiple Sclerosis	*p*-Value
	*n* = 250	*n* = 250	
	*n* (%)	*n* (%)	
≤40 years			
*SIRT1* rs3818292			
AA	102 (85.0)	105 (79.5)	0.337
AG	17 (14.2)	23 (17.4)	
GG	1 (0.8)	4 (3.0)	
A	221 (92.1)	233 (88.3)	0.151
G	19 (7.9)	31 (11.7)	
*SIRT1* rs3758391			
CC	68 (56.7) ^1^	51 (38.6) ^1^	**0.009**
CT	42 (35.0) ^2^	71 (53.8) ^2^	
TT	10 (8.3)	10 (7.6)	
C	178 (74.2)	173 (65.5)	0.035
T	62 (25.8)	91 (34.5)	
*SIRT1* rs7895833			
AA	86 (71.7)	78 (59.1)	0.040
AG	28 (23.3) ^3^	50 (37.9) ^3^	
GG	6 (5.0)	4 (3.0)	
A	200 (83.3)	206 (78.0)	0.133
G	40 (16.7)	58 (22.0)	

*p*-value—significance level and Bonferroni corrected significance level when *p* = 0.05/3; ^1^ *p* = 0.004, ^2^ *p* = 0.003, ^3^ *p* = 0.012; the bolded results indicate significant differences between the groups.

**Table 7:** Distribution of genotypes and alleles of *SIRT1* rs3818292, rs3758391, and rs7895833 in patients with multiple sclerosis and control groups between older participants.

Genotype/Allele	Control Group	Multiple Sclerosis	*p*-Value
	*n* = 250	*n* = 250	
	*n* (%)	*n* (%)	
>40 years			
*SIRT1* rs3818292			
AA	116 (89.2) ^1^	90 (76.3) ^1^	0.022
AG	12 (9.2)	22 (18.6)	
GG	2 (1.5)	6 (5.1)	
A	244 (93.8)	202 (85.6)	**0.002**
G	16 (6.2)	34 (14.4)	
*SIRT1* rs3758391			
CC	78 (60.0)	54 (45.8)	0.080
CT	45 (34.6)	55 (46.6)	
TT	7 (5.4)	9 (7.6)	
C	201 (77.3)	163 (69.1)	0.038
T	59 (22.7)	73 (30.9)	
*SIRT1* rs7895833			
AA	102 (78.5) ^2^	70 (59.3) ^2^	**0.002**
AG	25 (19.2)	37 (31.4)	
GG	3 (2.3)	11 (9.3)	
A	229 (88.1)	117 (75.0)	**<0.001**
G	31 (11.9)	59 (25.0)	

*p*-value—significance level and Bonferroni corrected the significance level when *p* = 0.05/3; ^1^ *p* = 0.007, ^2^ *p* = 0.001; the bolded results indicate significant differences between the groups.

**Table 8:** Binary logistic regression analysis of multiple sclerosis and control groups’ younger participants (<40 years).

Model	Genotype/Allele	OR (95% CI)	*p*-Value	AIC
≤40 years				
*SIRT1* rs3818292				
Co-dominant	AG vs. AA	1.314 (0.663–2.603)	0.433	350.472
	GG vs. AA	3.886 (0.427–35.357)	0.228	
Dominant	AG+GG vs. AA	1.457 (0.756–2.807)	0.260	349.491
Recessive	GG vs. AG+AA	3.719 (0.410–33.746)	0.243	349.091
Overdominant	AG vs. AA+GG	1.278 (0.646–2.529)	0.480	350.273
Allelic	G	1.485 (0.836–2.637)	0.177	348.895
*SIRT1* rs3758391				
Co-dominant	CT vs. CC	2.254 (1.331–3.443)	**0.002**	343.383
	TT vs. CC	1.333 (0.516–3.443)	0.552	
Dominant	CT+TT vs. CC	2.077 (1.256–3.435)	**0.004**	442.535
Recessive	TT vs. CT+CC	0.902 (0.362–2.248)	0.824	350.725
Overdominant	CT vs. CC+TT	2.162 (1.301–3.592)	**0.003**	341.735
Allelic	T	1.557 (1.041–2.329)	0.031	346.007
*SIRT1* rs7895833				
Co-dominant	AG vs. AA	1.969 (1.130–3.429)	0.017	246.263
	GG vs. AA	0.735 (0.200–2.702)	0.643	
Dominant	AG+GG vs. AA	1.751 (1.034–2.967)	0.037	346.370
Recessive	GG vs. AG+AA	0.594 (0.163–2.157)	0.428	350.133
Overdominant	AG vs. AA+GG	2.003 (1.156–3.473)	**0.013**	344.481
Allelic	G	1.404 (0.897–2.197)	0.137	348.526

OR—odds ratio, AIC—Akaike information criteria; the underlined AIC value indicates the best genetic model; CI—confidence interval; *p*-value—significance level; Bonferroni corrected significance level when *p* = 0.05/3; the bolded results indicate significant differences between the groups.

**Table 9:** Binary logistic regression analysis of multiple sclerosis and control groups’ older participants.

Model	Genotype/Allele	OR (95% CI)	*p*-Value	AIC
>40 years				
*SIRT1* rs3818292				
Co-dominant	AG vs. AA	2.363 (1.110–5.029)	0.026	339.433
	GG vs. AA	3.867 (0.762–19.613)	0.103	
Dominant	AG+GG vs. AA	2.578 (1.282–5.181)	**0.008**	337.754
Recessive	GG vs. AG+AA	3.429 (0.678–17.330)	0.136	342.641
Overdominant	AG vs. AA+GG	2.253 (1.061–4.786)	0.034	340.550
Allelic	G	2.177 (1.219–3.890)	**0.009**	337.543
*SIRT1* rs3758391				
Co-dominant	CT vs. CC	1.765 (1.044–2.984)	0.034	342.161
	TT vs. CC	1.857 (0.652–5.291)	0.246	
Dominant	CT+TT vs. CC	1.778 (1.074–2.944)	0.025	340.169
Recessive	TT vs. CT+CC	1.451 (0.523–4.027)	0.475	344.705
Overdominant	CT vs. CC+TT	1.649 (0.989–2.750)	0.055	341.517
Allelic	T	1.554 (1.028–2.348)	0.036	340.756
*SIRT1* rs7895833				
Co-dominant	AG vs. AA	2.157 (1.194–3.897)	**0.011**	334.615
	GG vs. AA	5.343 (1.438–19.848)	**0.012**	
Dominant	AG+GG vs. AA	2.498 (1.432–4.358)	**0.001**	334.487
Recessive	GG vs. AG+AA	4.352 (1.183–16.005)	0.027	339.230
Overdominant	AG vs. AA+GG	1.919 (1.069–3.442)	0.029	340.358
Allelic	G	2.225 (1.403–3.528)	**<0.001**	332.643

OR—odds ratio, AIC—Akaike information criteria; the underlined AIC value indicates the best genetic model; CI—confidence interval; *p*-value—significance level; Bonferroni corrected significance level when *p* = 0.05/3; the bolded results indicate significant differences between the groups.

**Table 10:** Genotype distribution and serum SIRT1 levels between patients with multiple sclerosis and control group.

Genotype	SIRT1 Level (pg/mL)		*p*-Value
	Control Median (IQR)	Multiple Sclerosis Median (IQR)	
rs3818292			
AA	0.239 (2.377)	1.245 (0.045)	**0.001**
AG	2.244 (1.805)	0.065 (−)	0.066 *
GG	-	-	-
rs3758391			
CC	1.898 (2.682)	0.536 (−)	0.751
CT	0.304 (2.770)	0.089 (0.037)	**<0.001**
TT	1.956 (2.707)	0.108 (−)	0.213 *
rs7895833			
AA	1.813 (2.812)	0.094 (0.054)	**0.002**
AG	1.872 (2.763)	0.089 (0.028)	0.004 *
GG	1.794 (−)	0.108 (−)	0.275

* Student *t*-test.; *p*-value—significance level; Bonferroni corrected significance level when *p* = 0.05/3; the bolded results indicate significant differences between the groups.

## Data Availability

All data relevant to the study are included in the article.
